# Scrotal hematoma caused by femoral artery puncture during ablation

**DOI:** 10.1002/joa3.70051

**Published:** 2025-03-24

**Authors:** Kosuke Muto, Tsukasa Naganuma, Hitoshi Mori, Yoshifumi Ikeda, Ritsushi Kato

**Affiliations:** ^1^ Department of Cardiology, International Medical Center Saitama Medical University Saitama Japan

**Keywords:** femoral artery puncture, scrotal hematoma

## Abstract

Scrotal hematoma is a rare complication of bleeding after femoral artery puncture. It often occurs without an associated inguinal hematoma, making it difficult to detect immediately after the procedure. Management of scrotal hematoma is usually conservative; however, surgical intervention or IVR may be necessary in cases of active bleeding.

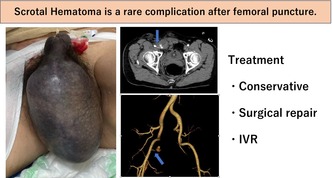

Complications of femoral artery puncture, such as inguinal hematoma, pseudoaneurysm, arteriovenous fistula, and retroperitoneal hematoma, are well known. While inguinal hematoma is a common occurrence after femoral artery puncture, scrotal hematoma is rare. Here, we report a case of scrotal hematoma following femoral artery puncture during ablation.

A 51‐year‐old male with a history of pulmonary vein isolation for atrial fibrillation (AF) 4 years ago presented to our hospital with frequent episodes of palpitations that had started a year prior. Electrocardiography showed a short RP' tachycardia with a heart rate of 175 bpm, leading to hospitalization for an electrophysiological study (EPS) and catheter ablation for suspected paroxysmal supraventricular tachycardia (PSVT).

EPS revealed that the tachycardia originated near the atrioventricular node, consistent with atrial tachycardia. Using the CARTO® system, we performed ablation at the anatomical slow pathway region and the tricuspid annulus, but the tachycardia did not terminate. Subsequently, we employed a transaortic approach and ablated the noncoronary cusp of the aortic valve. As a result, a 9Fr long sheath was inserted into the right femoral artery. Ultimately, the tachycardia could not be suppressed with ablation, and the procedure was concluded. The right inguinal puncture site was manually compressed for hemostasis before the patient was returned to the ward.

The morning after the ablation, the right inguinal puncture site remained compressed with gauze until that point. After the compression was released and the patient stood up and walked, he began experiencing pain in the right inguinal region and scrotum. Although no obvious inguinal hematoma was observed, significant scrotal swelling was noted (Figure 1). Suspecting bleeding from the punctured femoral artery, additional manual compression was applied for 30 min. However, since scrotal swelling worsened even after compression, contrast‐enhanced computed tomography (CT) was performed, revealing active bleeding from the right femoral artery and a hematoma extending from the right inguinal canal to the right scrotum (Figure 2).

**FIGURE 1:**
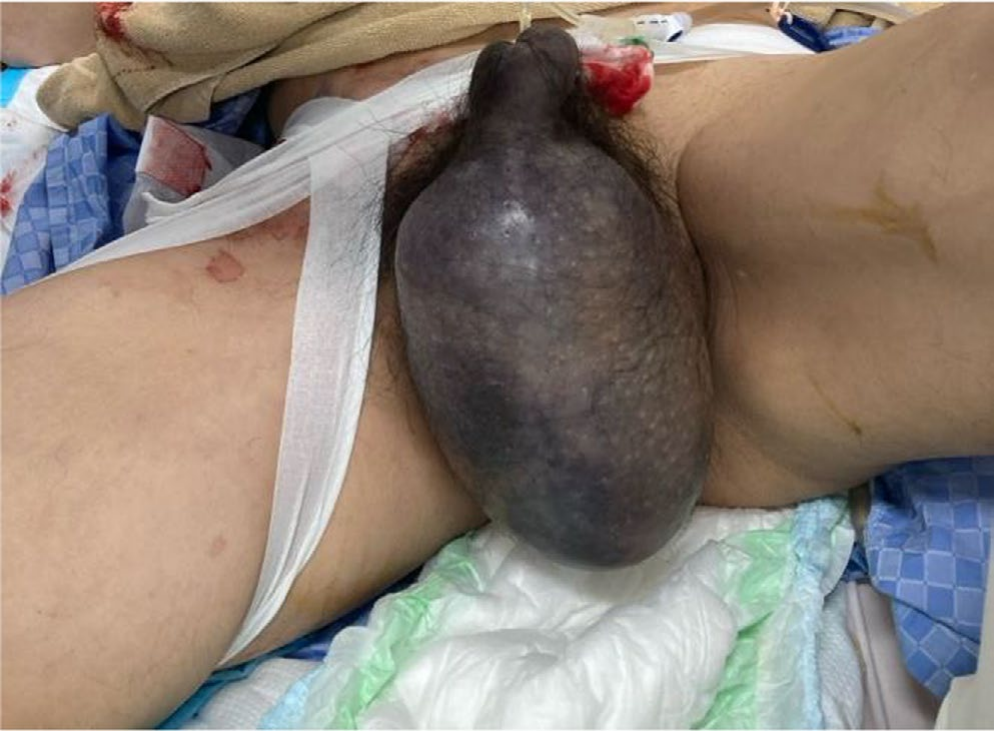
Significant scrotal hematoma on the day after ablation.

**FIGURE 2:**
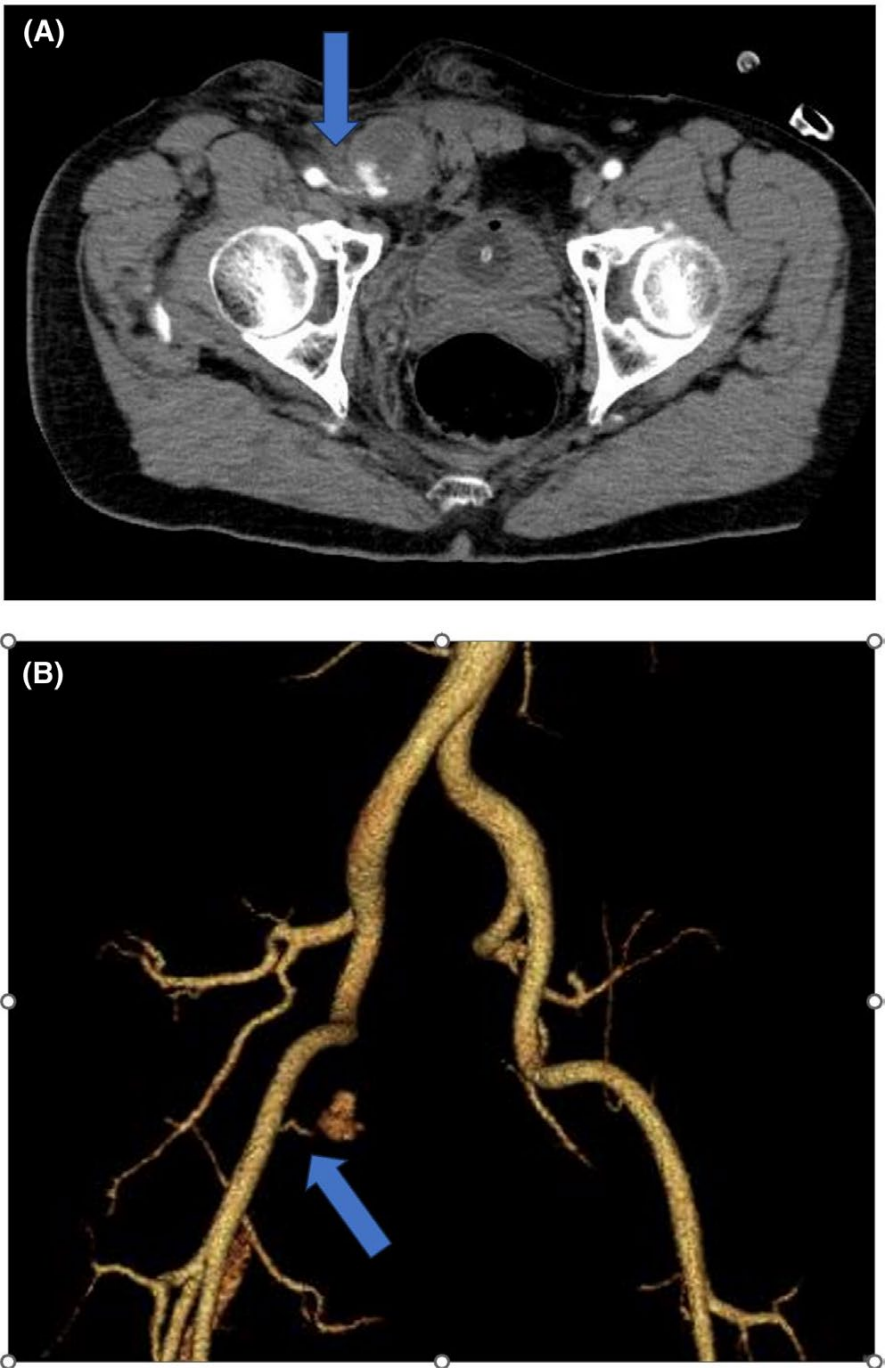
Contrast‐enhanced CT scan after manual compression. (A) The blue arrow indicates contrast leakage from the right femoral artery into the right inguinal canal. (B) The blue arrow indicates bleeding from a branch of the right femoral artery.

As manual hemostasis was deemed ineffective, surgical hemostasis was requested on the same day. The bleeding source was identified as a branch of the right common iliac artery, and clipping was performed at that site (Figure 3). Following surgical hemostasis, no further worsening of the scrotal hematoma was observed.

**FIGURE 3:**
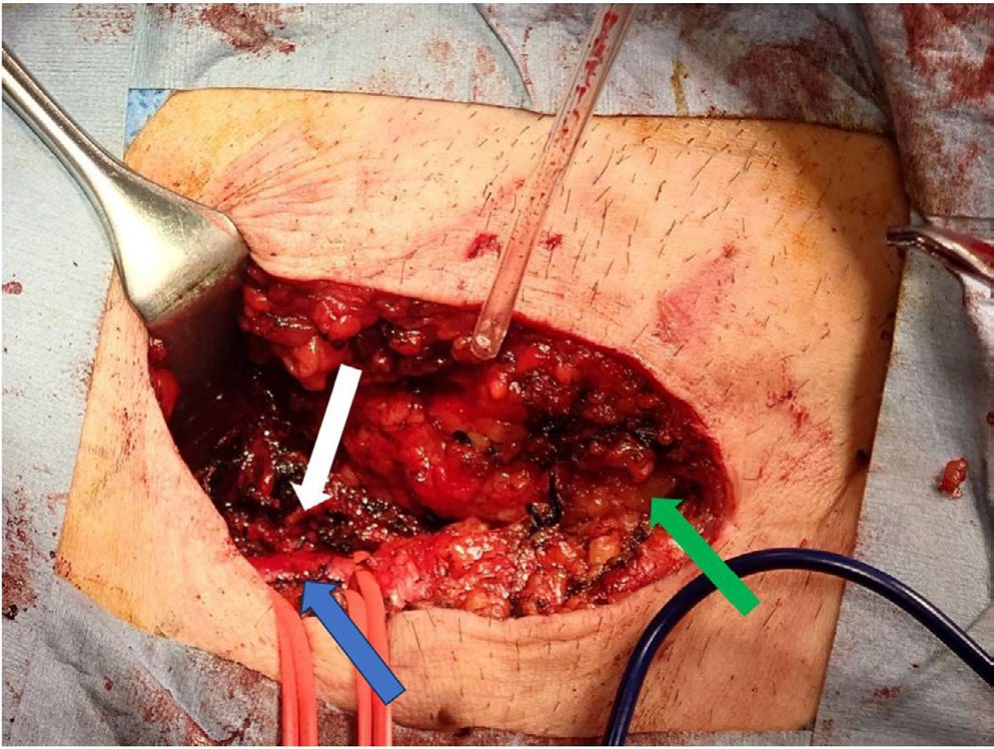
Intraoperative findings. The left side is cranial, and the right side is caudal. The blue arrow indicates the right femoral artery, the white arrow indicates the bleeding source, and the green arrow indicates the inguinal canal. The bleeding site was a branch from the right common iliac artery, and clipping was performed at this location.

Scrotal ultrasound revealed a right scrotal hematoma, but the right testis was normal in size and blood flow. After consultation with the urology department, conservative management with analgesics and compression therapy was chosen. Over time, the pain associated with the scrotal hematoma improved, and the hematoma gradually decreased in size, allowing for discharge home.

A scrotal hematoma can occur following testicular torsion, adrenal hemorrhage, or birth trauma, and cases of idiopathic scrotal hematoma have also been reported.^1^ However, it is rare for a scrotal hematoma to appear as a bleeding complication after femoral artery puncture. In cases where bleeding occurs above the inguinal ligament, such as retroperitoneal hemorrhage or bleeding from the inferior epigastric artery, blood may spread through the preperitoneal space, descend along the spermatic cord and inguinal canal, and lead to an inguinal and scrotal hematoma.^2^

The incidence of major bleeding complications after the cardiac catheterization ranges from 2% to 6%; however, scrotal hematoma is a rare complication.^3^ The risk of access site bleeding is higher in female gender, older age, obese patients, renal impairment, interventional procedures as compared to diagnostic procedures, use of anticoagulation, and increased sheath size.^3^

At our hospital, an arterial pressure line is secured via the inguinal approach without ultrasound guidance for hemodynamic monitoring during ablation procedures. It is reported that using ultrasonography reduces the risk of venipuncture; however, it does not reduce bleeding or vascular complications.^3^ Then, the inguinal approach without ultrasound guidance is chosen to shorten the procedure time rather than the radial artery approach.

In this case, the source of bleeding was not the main trunk of the right common iliac artery but a peripheral branch running medially from the right common iliac artery. It was suspected that this branch was injured when the arterial kit inserted in the right groin was replaced with a 9Fr long sheath for catheter ablation of the aortic noncoronary cusp via the transaortic approach. Despite being manipulated under fluoroscopic guidance, the wire exhibited good maneuverability without any tortuosity or stenosis in the femoral artery, which likely made it easier to enter the side branches. More careful wire manipulation may have prevented the vascular complication.

The treatment of scrotal hematoma generally involves conservative management, including the use of analgesics, blood transfusion, bed rest, scrotal elevation, and observation. Surgical intervention or Interventional Radiology (IVR) is required only when there is active bleeding because of vascular injury, progressive enlargement of the hematoma, or impaired blood flow to the testes.^3^ In this case, although the patient remained hemodynamically stable with only a mild decrease in hemoglobin levels, active bleeding and expansion of the scrotal hematoma were observed, necessitating surgical repair or IVR. However, in the case of IVR, a femoral artery puncture approach was required, which could potentially lead to rebleeding complications. In this case, the patient's hemodynamics were stable, and they were able to tolerate surgery; therefore, surgical hemostasis was chosen. On the other hand, if the patient had unstable hemodynamics and lacked surgical tolerance, IVR could be considered as an alternative.

## CONFLICT OF INTEREST STATEMENT

The authors report no conflicts of interest.

## ETHICS STATEMENT

Informed consents were obtained from the patients to publish the case report.
